# The Nutritional Geometry of Resource Scarcity: Effects of Lean Seasons and Habitat Disturbance on Nutrient Intakes and Balancing in Wild Sifakas

**DOI:** 10.1371/journal.pone.0128046

**Published:** 2015-06-10

**Authors:** Mitchell T. Irwin, Jean-Luc Raharison, David R. Raubenheimer, Colin A. Chapman, Jessica M. Rothman

**Affiliations:** 1 Department of Anthropology, Northern Illinois University, DeKalb, Illinois, United States of America; 2 SADABE Madagascar, Antananarivo, Madagascar; 3 Department of Animal Biology, University of Antananarivo, Antananarivo, Madagascar; 4 Charles Perkins Centre, Faculty of Veterinary Science, The University of Sydney, Sydney, New South Wales, Australia; 5 School of Biological Sciences, The University of Sydney, Sydney, New South Wales, Australia; 6 Department of Anthropology, McGill University, Montreal, Quebec, Canada; 7 McGill School of Environment, McGill University, Montreal, Quebec, Canada; 8 Wildlife Conservation Society, Bronx, New York, United States of America; 9 Department of Anthropology, Hunter College, City University of New York, New York, New York, United States of America; 10 New York Consortium in Evolutionary Primatology (NYCEP), New York, New York, United States of America; Midwestern University & Arizona State University, UNITED STATES

## Abstract

Animals experience spatial and temporal variation in food and nutrient supply, which may cause deviations from optimal nutrient intakes in both absolute amounts (meeting nutrient requirements) and proportions (nutrient balancing). Recent research has used the geometric framework for nutrition to obtain an improved understanding of how animals respond to these nutritional constraints, among them free-ranging primates including spider monkeys and gorillas. We used this framework to examine macronutrient intakes and nutrient balancing in sifakas (*Propithecus diadema*) at Tsinjoarivo, Madagascar, in order to quantify how these vary across seasons and across habitats with varying degrees of anthropogenic disturbance. Groups in intact habitat experience lean season decreases in frugivory, amounts of food ingested, and nutrient intakes, yet preserve remarkably constant proportions of dietary macronutrients, with the proportional contribution of protein to the diet being highly consistent. Sifakas in disturbed habitat resemble intact forest groups in the relative contribution of dietary macronutrients, but experience less seasonality: all groups’ diets converge in the lean season, but disturbed forest groups largely fail to experience abundant season improvements in food intake or nutritional outcomes. These results suggest that: (1) lemurs experience seasonality by maintaining nutrient balance at the expense of calories ingested, which contrasts with earlier studies of spider monkeys and gorillas, (2) abundant season foods should be the target of habitat management, even though mortality might be concentrated in the lean season, and (3) primates’ within-group competitive landscapes, which contribute to variation in social organization, may vary in complex ways across habitats and seasons.

## Introduction

One of the most fundamental ecological challenges animals face is a lack of suitable food, and a large proportion of most species’ anatomical, physiological, and behavioral traits can be viewed as adaptations to finding, acquiring and processing food. Despite this, little is known about how pervasive food stress is for wild herbivores, with some evidence that herbivores are not generally limited by food availability (the "green world paradox") [1]. Aside from case studies illustrating extreme shortage such as droughts [2, 3], and a general consensus that food stress should exist when populations approach carrying capacity or in environments without predators, little is known about the variation in nutritional inputs and food stress across space and time.

A logical first step in understanding an animal’s nutritional challenges is to quantify its average daily intake of energy and macronutrients, through measuring food chemistry, feeding time, and intake rates to determine if nutrient intake exceeds estimated requirements [4–8]. However, in attempting to understand what makes one diet “higher quality” than another, considering individual requirements in isolation falls short, for two reasons. First, to some extent, macronutrients are interchangeable—especially when used as an energy source—making it problematic to discuss “requirements” in isolation. Second, targets are not simply minima. Overingestion of a nutrient (eating more than required) incurs a metabolic cost, as the animal mobilizes and excretes the excess; the magnitude of this cost depends on that animal’s adaptations to such “overshoots”. Other components of foods are actually undesirable, such as plant secondary metabolites (PSMs) [9]; for these, animals presumably seek to minimize intakes. It has become clear that an integrated approach, which simultaneously assesses shortfalls and surpluses for multiple nutritional variables, is needed. The development of the “geometric framework of nutrition” [10, 11], a multivariate approach in which two or more nutrients are used to form a geometric space within which nutritional targets and outcomes can be quantified, shows promise in understanding strategies used when food is scarce. This multidimensional representation of nutritional landscapes and outcomes allows researchers to better untangle nutritional targets, and the constrained responses (“rules of compromise”) when targets are unattainable. Using nutritional geometry in assessing nutritional challenges appears increasingly appropriate, given the growing evidence that the proportional composition of diet has health consequences separate from the effects of simple nutrient or energy density [11, 12].

Although many dietary strategies are theoretically possible, empirical work has so far revealed the existence of five potential strategies used by different species [5]. Two of these are maximization rules (for energy and protein) [13], the third is minimization of PSMs [9, 14], the fourth is the “regulation” of fiber intake [15, 16], and the fifth is nutrient balancing, as explored by the geometric framework [11], whereby macronutrient proportions are optimized rather than absolute amounts. All of these have empirical support; in many cases one nutrient or toxin may be “limiting” and dominate foraging choices, in others a balancing approach may emerge. What remains unclear is what factors determine which regulation strategies apply to specific populations (and what proportion of the variation is due to phylogenetic/physiological differences rather than environmental differences).

Seasonal variation in primate diets is well-documented [17]; many primates exploit preferred foods during the hotter, more rainy season, and switch in the drier, cooler “lean season” to fallback foods, whose consumption is negatively correlated with the availability of preferred foods [18–20]. The degree of seasonality varies, and its challenges can relate either to the quantity (availability) or quality of foods [20]. Lean season reductions in food “quality” can be manifested in lower density of nutrients and energy (less nutritional payoff per gram of food). Alternatively, this can reflect other properties that limit what primates can ingest or absorb: physical properties can reduce ingestion or absorption efficiency, fiber content may limit gut transit time, and PSMs can reduce amounts ingested and/or its absorption efficiency. Primates likely use both behavioral adaptations (including food-switching) and physiological responses such as changes in gut morphology, function, and absorption efficiency to reduce the degree to which they miss nutritional targets [17]. It is also possible that primates use seasonal mass change (including fat storage) and reductions in energy expenditure (in the extreme, hibernation) to balance short-term deficiencies against surpluses in other seasons [8, 21, 22].

To date, few studies have quantified the degree of seasonal variation in primates’ nutrient intakes, but available evidence suggests considerable variability. Mountain gorillas experience less than 20% seasonal reduction in energy intakes [7], while sifakas experience a 74% reduction [20], chimpanzees a 46% reduction, and orangutans an 85–90% reduction [23, 24]. More research is needed to understand the ways in which the lean season forces them to “miss” nutritional targets, and what their rules of compromise are.

Fewer still are studies that have investigated differences in nutrient prioritization across seasons using the geometric framework. Felton et al. [25] found that spider monkeys (*Ateles chamek*) use “protein-leverage”, optimizing available protein (AP) intake while allowing the intake of non-protein energy (NPE: carbohydrates and fat) to vary widely, apparently consuming these in excess during some seasons. This is similar to experimental results examining human diets [26, 27]. Rothman et al. [28] found a different pattern in mountain gorillas (*Gorilla beringei*): NPE intake (carbohydrates, fat, and fiber) was constant across the year, while AP intake varied (with apparent surpluses in higher-folivory seasons). Both studies made the simplifying assumption that the more tightly-regulated nutrient is limiting, and therefore driving foraging choices, while the other is less tightly-regulated and often consumed in excess; they have constrained choice because nutrients are packaged in fixed ratios. This interspecific difference may reflect food characteristics—gorilla foods have high protein content while spider monkey foods are largely lower-protein, carbohydrate-rich fruits.

An extension of this research to which the geometric framework can be applied is how animals experience and mitigate food scarcity more broadly: in other words, systemic differences rather than cyclically-occurring seasonal challenges. Systemic differences can exist among habitats (spatial heterogeneity), or can be progressive: a longer-term within-site change in food availability and/or quality, for example resulting from deforestation, habitat fragmentation, selective logging, or climate change. Several studies have documented dietary change in response to these factors [29–32], but relatively few have documented nutritional properties of foods. Howler monkeys (*Alouatta pigra*) increased mature leaf consumption and preferred mineral-rich foods following a cyclone [33], and brown lemurs (*Eulemur collaris*) in a highly-disturbed littoral forest increased mature leaf consumption and used primary fruit resources that were higher in lipids and lower in carbohydrates compared to a less-disturbed forest nearby [34]. Red-tailed monkeys (*Cercopithecus ascanius*) in heavily logged forest increased consumption of young and mature leaves, petioles and unripe fruit, and had reduced estimated daily intake (derived from scan samples) of protein, lipids and key minerals but higher estimated intakes of sugars [35]. To our knowledge no primate study has yet quantified daily intakes across a habitat disturbance gradient using focal animal data. It is important to remember that “disturbance” does not necessarily have a negative impact; for example Ganzhorn [36] documented increased protein concentration in leaves, increased fruiting, and higher encounter rates for lemurs in mildly logged forest. Even when dietary shifts are observed in disturbed habitat, underlying nutritional intakes could be higher, lower or equivalent.

The fact that so little is known about nutritional heterogeneity across habitats of varying disturbance leaves us often ill-equipped to prioritize populations for conservation efforts, and design concrete management strategies. Yet there is little doubt that primates’ dietary shifts have potentially severe consequences for individuals and populations, potentially depressing reproductive rates and/or causing extirpations [37, 38]. An increased understanding of both “natural” seasonal changes in nutritional intake, and additional changes resulting from habitat alteration, will certainly improve conservation planning.

We use the geometric framework of nutrition [11] to describe energy and macronutrient intake in diademed sifakas (*Propithecus diadema*), living in a seasonal rainforest in Madagascar. Previous study [20, 29] documented lean season reductions in energy and macronutrient intakes for two groups living in relatively intact forest, which resulted from reductions in food intakes rather than reduced nutrient concentrations in foods. Here we present new data with three additional groups from degraded habitat, and examine the nutritional manifestations of both seasonal and systemic food scarcity. First, we examine seasonality and inter-group differences in foraging effort, degree of frugivory, and daily intakes. Second, we quantify the nutritional consequences of seasonality and habitat variation simultaneously in a geometric framework (variation in the relative contribution of macronutrients to daily diets). Third, we examine evidence for rules of compromise in both habitats; in other words, do sifakas prioritize some macronutrients more than others when constrained from reaching their intake target? Because of the phenology data indicating greater food availability, we expected higher nutritional intakes in the abundant season, and because dietary overlap among sites is highest in the lean season [29] we expected greatest inter-site divergence in the abundant season. We made no specific predictions about nutrient ratios as we felt this was precluded by a lack of comparative data.

## Materials and Methods

### Study Site and Subjects

Tsinjoarivo forest is a mid-altitude forest in eastern central Madagascar. Diademed sifakas (*Propithecus diadema*) at Tsinjoarivo have been studied since 2002 [29, 39, 40]. We studied five groups from June 2006 to July 2007: two in continuous, relatively undisturbed habitat at Vatateza (“CONT” groups; 19°43.250S, 47°51.410E; 1,396 m) and three in fragmented, disturbed habitat at Mahatsinjo (“FRAG” groups; 19°40.940S, 47°45.460E; 1,590 m); some data from CONT groups was previously presented in [20]. Botanical data [41] revealed that cumulative basal area/ha of trees >5 cm DBH was a useful correlate of habitat disturbance, as removal of large trees seems to have caused a significant reduction in this variable which has not yet been recovered. CONT groups had high values, FRAG4 was intermediate, FRAG2 was low and FRAG3 was very low (Table 1). This pattern was not driven by regrowth of small trees following disturbance: >80% of basal area in all plots was in trees >10 cm DBH, and basal area/ha of trees >10 cm DBH showed the same ordering.

**Table 1:** Habitat characteristics, sample size, average daily energy and protein intakes, and ratio of available protein to non-protein energy (fat, carbohydrate and NDF) for five sifaka groups at Tsinjoarivo, Madagascar.

Group	Habitat Basal Area per hectare / Disturbance Level	Seasons Sampled	n (days)	Daily Energy Intake (kJ)	Energy per metabolic body mass (kJ●BM_kg_^-0.762^●day^-1^)	Daily Available Protein Intake (g)	Available Protein Intake (g●BM_kg_^-1^●day^-1^)
**CONT1**	39.6 / low	1–5	106	3667±3080	1132	18.1±14.1	3.9
**CONT2**	44.7 / low	1–5	91	4792±4267	1350	23.2±19.7	4.4
**FRAG2**	17.4 / high	1–5	87	2235±1273	677	14.6±7.0	3.0
**FRAG3**	9.4 / very high	1	10	1326±373	407	9.4±3.2	2.0
**FRAG4**	22.8 / moderate	2–5	69	3643±3810	1084	16.1±11.2	3.3
**TOTAL**			363	3537±3367	1049	17.9±14.2	3.6

The diet was quantified by Irwin (2008b): sifakas’ annual diet in terms of plant parts is relatively consistent across groups (53% of feeding time on foliage 24% on fruits, 7% on seeds, 15% on flowers), with an abundant season (November to mid-April) emphasis on fruits and seeds and a lean season (mid-April through October) emphasis on leaves and flowers, largely from the mistletoe *Bakerella clavata*, a fallback food. FRAG groups have lower dietary diversity and differ from CONT groups in that their fruits derive largely from mistletoe rather than canopy trees.

All animal work was carried out in accordance with the Animal Care and Use Guidelines Document of the American Society of Mammalogists, was approved by McGill University’s Animal Care Committee (AUP#5231), and adhered to the legal requirements of Madagascar. Focal animals were captured (after pre-habituation) at close range using a blowgun loaded with Pneu-dart 9mm disposable nonbarbed darts [40] containing tiletamine/zolazepam (Telazol, Fort Dodge Animal Health, Overland Park, Kansas 66225; 25 mg/kg) [40]. Following physical examination, morphometric data collection, blood collection and collaring for field identification, a balanced electrolyte solution equivalent to blood volume collected was administered subcutaneously and animals recovered in burlap sacks before re-release at the capture site or near group-mates. Research permits were issued in Madagascar by CAFF/CORE and the Ministry of Environment, Forests and Tourism (#0156-08/MEEFT/SG/DGEEF/DSAP/SSE).

### Observational Data

We collected data in 12 data collection periods, each with 11–21 days of observation. For simplicity, these periods were categorized into 5 seasons: (16 June-14 August 2006; periods 1–2), 2 (22 October–19 December 2006; periods 3–5), 3 (27 January–14 April 2007; periods 6–8), 4 (27 April–2 June 2007; periods 9–10), and 5 (18 June–26 July 2007; periods 11–12). Seasons 1, 4 and 5 represent lower than average temperature and rainfall, while seasons 2 and 3 experience higher than average temperature and rainfall; detailed description of these seasons is provided in [29, 42]. We refer to seasons 1, 4 and 5 as the lean season and seasons 2–3 as the abundant season (the latter’s percentage-based phenology scores are 227% higher for fruits and 62% higher for young leaves [29, 42]). Sifakas give birth in June or July (Seasons 1 and 5), lactate until roughly January (Seasons 1–3), mate in December (between seasons 2 and 3) and gestate between December and June/July (seasons 3–4). The CONT2 and FRAG2 adult females lactated during the first half of the study but were not gestating during the second half; CONT1 and MAHA4 adult females lactated during the first half and gestated during the second half; the MAHA3 adult female did not give birth in 2006 and died shortly after the birth season.

Data were collected on all adults and animals ≥ 2 years old at the beginning of the study (2-year-olds are roughly 70% adult body mass) during all-day focal-animal follows, led by MTI and/or local research assistants. Body mass data were available for most animals from captures within 2 years of this study. When observers returned to groups on subsequent days animals were almost always in the same sleeping sites, suggesting that they did not feed at night. For each feeding bout, we recorded start and stop time, plant part and species consumed. Bouts were stopped when a pause exceeded 10 seconds. Soil feeding was recorded, but not included in this analysis (0.3% of feeding time). Our sample includes 363 focal-animal days, 18,253 feeding bouts and 1090 feeding hours split across 18 individuals (CONT1: 5; CONT2: 4). Animals were well-habituated and were rarely out of sight (0.2% of 5-min instantaneous records).

When observation conditions allowed, we recorded intake rates in one of two ways. For rapidly-consumed items, such as young leaves or flowers, we sampled one-minute intervals within feeding bouts, recording the number of units consumed (some intervals were shorter, when the bout ended before one minute had passed). For larger items (usually fruits and seeds), we recorded start and stop time for each item, or a subset of items, within bouts. We amassed 16,565 intake records totaling 283 hr across the five groups.

### Sample Collection, Chemical Analyses and Nutrient Calculations

We collected 134 samples of sifaka foods representing 58 plant species and 87 species—plant part combinations. Parts sampled were flower buds, flowers, fruit without seed (when seeds were spit or dropped), fruit with seed, seed, young leaves and distal growing shoots. Where possible, samples were collected from plants actually fed on; failing that we selected nearby conspecific plants as similar as possible to those used (in terms of size, phenological state and microhabitat). Samples were processed in the same way as by sifakas. Samples were dried in trays inside a tent pitched in direct sun.

Nutritional analyses followed Rothman et al. (2012), and included assays for crude protein (using a Leco FP-528 combustion analyzer), water-soluble carbohydrates (phenol-sulfuric acid assay), fat (ether extract), all three fiber fractions (neutral detergent fiber, acid detergent fiber, and lignin; measured by sequential analysis using an A200 Fiber Analyzer, ANKOM, Macedon, NY), and ash. Available protein was calculated by subtracting acid detergent-indigestible protein (ADICP) from crude protein, and “Total Non-structural Carbohydrates” (TNC) was calculated as:

TNC=100−(Fat+(CP−ADICP)+Ash+NDF)

We estimated energy content of foods (kJ/g), through summing the physiological fuel values of their components [43], as follows:

$$E=\left(AP\times 16.736\right)+\left(TNC\times 16.736\right)+\left(Fat\times 37.656\right)+\left(Dig_{cell}\times \left(ADF-Lignin\right)\times 12.552\right)+\left(Dig_{hc}\times \left(NDF-ADF\right)\times 12.552\right)$$

where E = energy content (kJ/g); Dig_cell_ = 0.3911, and Dig_hc_ = 0.5197. Following Altmann [4], we used the following formula to calculate daily nutrient intakes for focal animals:

$$DI_{y}=\overset{B}{\underset{i=1}{\sum }}D_{i}\;\times \;R_{x}\;\times \;M_{x}\;\times \;C_{x}\;\times \;Q_{x,y}$$

where DI = daily intake of y (expressed in grams for nutrients, kCal for energy), B = number of feeding bouts, D_i_ = duration of feeding bout i (sec), R_x_ = average intake rate (units/sec) for food x (plant part / species combination), M_x_ = mass per intake unit (g/unit dry matter) for food x, C_x_ = intake conversion factor for food x (for *Bakerella* flowers and *Salacia madagascariensis* seeds), and Q_x,y_ = nutrient concentration or energy density of macronutrient y in food x (percent of dry matter for nutrients, kJ/g for energy). For detailed descriptions of analytical procedures and calculations, including fiber digestibilities, see [20]; raw data are found in S1 Table.

In calculating daily intakes we used average intake rates (pooled across individuals and sites), which is potentially misleading if differences across individuals and sites occur; however, using more specific intake rates would lead to smaller sample sizes, yielding estimates potentially farther from true values. Analysis showed: (1) for 76 foods consumed at both sites, a near-equal likelihood of having higher mean intake rate in CONT groups (41 foods) vs. FRAG groups (35 foods; binomial test, *P* = 0.8); (2) for 119 foods consumed by adults of both sexes, females (58 foods) and males (61 foods; binomial test, *P* = 0.6) were equally likely to have higher intake rates, and (3) for 119 foods consumed by adults and immatures, immatures tended to have lower intake rates (75 of 119 foods; binomial test, *P* = 0.003). However, we still chose to use pooled intake rates; even for the immature-adult difference, the average percentage difference among these foods (1.6%) was small relative to the error that could be introduced by using smaller sample sizes.

In total, nutritional data were available for foods representing 76% of feeding time for CONT groups and 84% of feeding time for FRAG groups. Because of the high dietary diversity, we were unable to sample all foods; we made an effort to preferentially sample those foods most important to overall diet. When nutritional information for a certain food was unavailable, we substituted data from other samples representing the same plant part and stage. If congeners had been sampled, these values were used; when no congeners were in the diet, we used the average for all species for that plant part/stage.

### Analyses

We compared eleven intake variables (feeding time, dry mass ingested, proportion of diet from fruit/seed, energy, available protein, Fat, TNC, NDF, ADF, Lignin and AP:NPE) using linear mixed models (LMM; SPSS Statistics, v. 20.0). Rather than using all daily intake measures, we used individual averages within each of the 12 data collection periods, which created a more balanced data structure (averages were based on 1–5 days). The data matrix included 18 individuals and 12 time periods, with 152 of 216 cells populated; gaps were due to animal emigrations and the shift from FRAG3 to FRAG4 (see above). The models included three fixed factors (group, data collection period, interaction), one random factor (individual nested within group, intercept only, variance components covariation structure), and a repeated term (diagonal covariation structure) to account for the repeated measures of individuals. Because the change in intake variables across seasons was complex and non-linear, we treated period as a categorical factor; this conservative approach increases model parameterization by estimating population-wide means for each period. This model structure was selected from several evaluated model structures using -2 REML log-likelihood scores [44]. Model fit was evaluated using three diagnostics: Q-Q plots, Kolmogorov-Smirnov Tests for normality of residuals, and residual vs. fitted plots. One dependent variable (proportion of diet from fruit/seed) was arcsine-transformed; for another (available protein) the model failed to converge, but converged after the use of a log transformation. Because of good diagnostic fit AP:NPE ratio was not transformed. A square-root transform was considered for all other variables; this only marginally improved residual normality and was not used (when residual distributions deviated from normality it was due to leptokurtosis, not left- or right-skew); raw and square-root transformed data yielded identical results for fixed factors. The significant group x period interaction makes assessing group-specific effects problematic; instead estimated marginal means are presented (which roughly represent each group’s “average” intake over the year) with pairwise differences evaluated (LSD method). Plots combine data collection periods (n = 12) into seasons (n = 5) for clarity of presentation.

Although sifakas are typically considered folivores, our dataset showed only 9.1% of macronutrient calories were derived from fiber (range across seasons 8.0–10.2%). We therefore used 3-variable right-angled mixture triangles to examine percentage contributions to the diet made by available protein, carbohydrates (TNC) and fat [45]. We used bivariate plots of available protein vs. NPE (TNC+Fat+NDF) following Rothman et al. [28].

## Results

### Foraging and Intakes

Average energy and protein intakes (both raw and scaled to body mass) were highest in the CONT groups (which lived in the least-disturbed habitat), lowest in the groups FRAG2 and FRAG3 (most-disturbed habitat), and intermediate in FRAG4 (moderate disturbance; Table 1).

**Fig 1:**
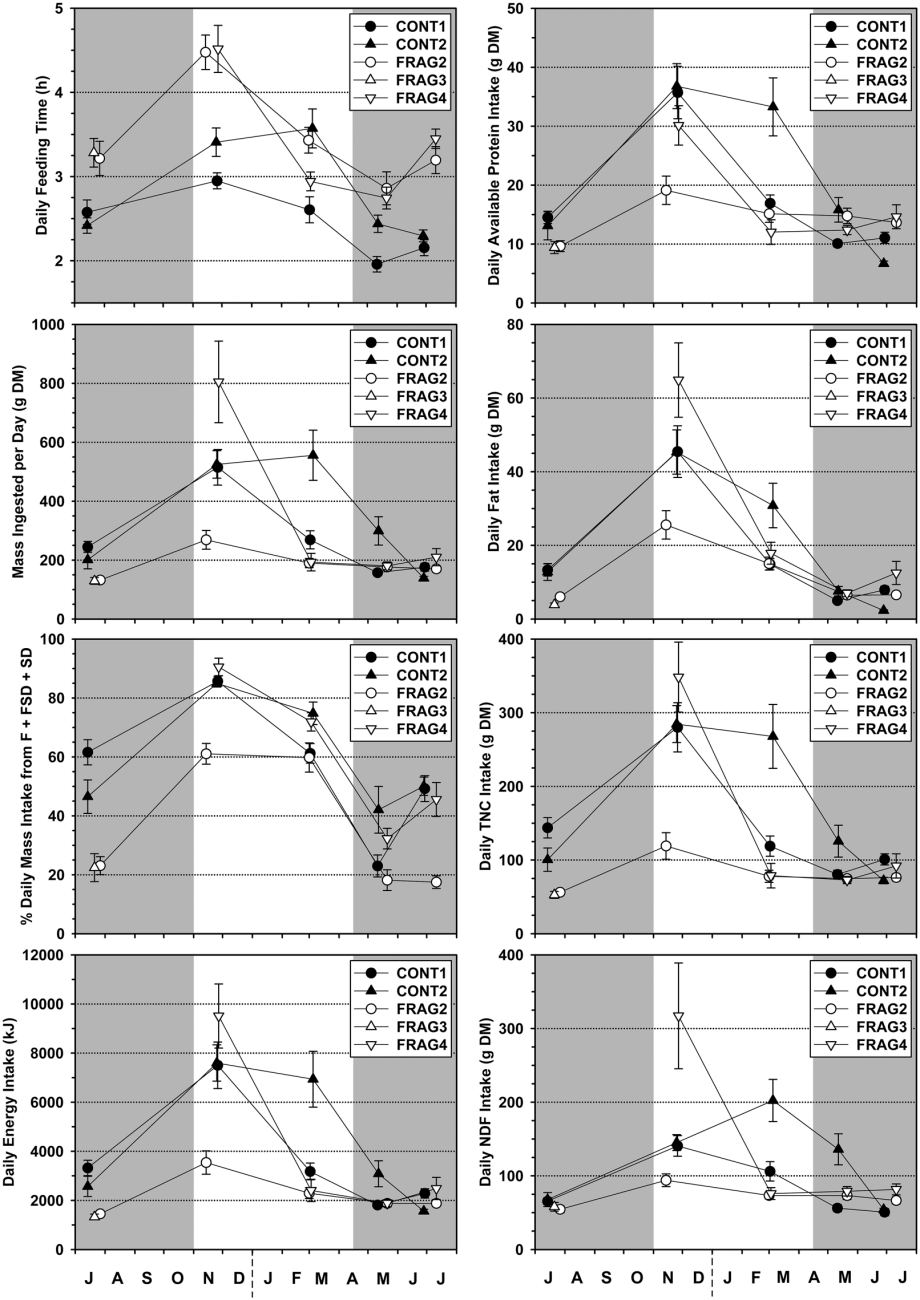
Foraging and intake variables for five sifaka groups across five seasons at Tsinjoarivo, Madagascar. The lean season is indicated by grey shading, error bars represent standard error.

All groups increased feeding time in the abundant season. FRAG groups spent more time feeding per day than CONT groups (except CONT2 in season 3), yet their mass ingested was less, especially in the abundant season (except FRAG4 in season 2; Fig 1, Table 2). CONT groups’ mass intakes increased from 139–300 g in the lean season to 269–556 g in the abundant season; FRAG2 showed a smaller increase, from 132–176 g in the lean season to 190–269 g in the abundant season but FRAG4 was the most variable, with a very high value in season 2 (800 g) and extremely low values in seasons 3–5. All groups were more frugivorous (higher contribution of fruit/seed to mass intake) in the abundant season; FRAG2 and FRAG3 were less frugivorous than CONT groups, while FRAG4 was similar (Fig 1). For CONT groups, energy, macronutrient and fiber intakes were consistently higher in the abundant season and lower in the lean season, often showing a three- to four-fold difference. FRAG groups resembled CONT groups in lean season intakes, but saw only modest abundant season increases (except for FRAG4 in season 2), largely mirroring the dry mass intakes. LMM results revealed significant effects of group and season, and group*season interaction, for all foraging and intake variables tested (Table 2). Energy intake scaled to metabolic body mass and available protein scaled to body mass follow similar patterns, with lower values and less seasonal variation in FRAG groups (Table 3).

**Table 2:** Linear Mixed Model results for 10 daily intake variables for five sifaka groups at Tsinjoarivo, Madagascar; data are individual lemurs’ average scores within each of 12 data collection periods (n = 152).

Intake Variable	Effect of:^1^			Estimated Marginal Means and Intergroup Differences^2^				
	Group	Period (1–12)	Group*Period	CONT1	CONT2	FRAG2	FRAG3	FRAG4
**Feeding time (sec)**	6.2 (0.004)	20.4 (<0.001)	5.9 (<0.001)	9067	10816^a^	12449^a^	11835^a^	11953^a^
**Dry mass (g)**	19.2 (<0.001)	25.3 (<0.001)	20.9 (<0.001)	283^b^	373	192^a^	128^a^	300^b^
**Proportion of mass from fruit/seed (%)** ^**3**^	11.0 (<0.001)	136.4 (<0.001)	13.0 (<0.001)	59.1^b^	61.9^b^	39.2^a^	22.0^a^	63.6^b^
**Energy (kJ)**	19.9 (<0.001)	25.4 (<0.001)	18.1 (<0.001)	3759^b^	4753	2279^a^	1309^a^	3561^b^
**Available Protein (g)** ^**4**^	3.3 (0.03)	45.6 (<0.001)	13.1 (<0.001)	15.2^ab^	17.3^a^	13.4^b^	9.2^b^	13.9^b^
**Fat (g)**	6.2 (0.001)	34.9 (<0.001)	7.7 (<0.001)	18.4^a^	21.9^a^	13.3	3.9	22.3^a^
**TNC (g)**	26.4 (<0.001)	21.9 (<0.001)	18.2 (<0.001)	148.7^b^	185.5	81.5^a^	51.8^a^	128.4^b^
**NDF (g)**	14.2 (<0.001)	30.4 (<0.001)	20.2 (<0.001)	88.0^a^	129.7^c^	74.2^ab^	57.5^b^	118.4^c^
**ADF (g)**	8.6 (<0.001)	19.3 (<0.001)	10.5 (<0.001)	60.4^a^	84.4^b^	60.6^a^	48.1^a^	95.5^b^
**Lignin (g)**	9.3 (<0.001)	19.8 (<0.001)	10.8 (<0.001)	36.8^a^	51.0	38.3^a^	28.9^a^	63.6
**AP:NPE** ^**5**^	31.2 (<0.001)	25.1 (<0.001)	7.3 (<0.001)	0.094^a^	0.092^a^	0.123^b^	0.134^b^	0.103

^1^ significance tests were not Bonferroni-corrected, but only 1.65 factors would be expected to be significant due to chance alone.

^2^ shared superscripts (a,b,c) within a row indicate EM means that are not significantly different.

^3^ percentage data were arcsine-transformed (EM means back-transformed into raw data).

^4^ model using raw data failed to converge; therefore data were log-transformed to improve normality (EM means back-transformed into raw data).

^5^ model using raw data converged but with non-positive final Hessian matrix; this did not improve with log or arcsine transformation, therefore model fit should be treated as preliminary.

**Table 3:** Seasonal variation in the daily energy and nutrient intakes (mean±SD) of diademed sifakas at Tsinjoarivo, Madagascar, scaled to metabolic body mass and body mass, respectively.

Group	Energy per metabolic body mass (kJ●BM_kg_^-0.762^●day^-1^) and Available Protein Intake (g●BM_kg_^-1^●day^-1^)				
	Season 1	Season 2	Season 3	Season 4	Season 5
**CONT1**	1017 ± 374	2311 ± 1454	985 ± 608	561 ± 171	709 ± 276
	3.07 ± 0.92	7.61 ± 4.80	3.63 ± 1.79	2.16 ± 0.60	2.38 ± 0.96
**CONT2**	714 ± 401	2132 ± 919	1957 ± 1600	879 ± 618	444 ± 99
	2.44 ± 1.58	6.96 ± 3.24	6.34 ± 4.67	3.04 ± 1.68	1.29 ± 0.36
**FRAG2**	443 ± 123	1079 ± 608	689 ± 297	564 ± 155	572 ± 158
	2.02 ± 0.75	3.98 ± 2.06	3.12 ± 1.36	3.03 ± 0.97	2.86 ± 0.93
**FRAG3**	407 ± 114	--	--	--	--
	2.00 ± 0.70	--	--	--	--
**FRAG4**	--	2799 ± 1457	713 ± 643	565 ± 188	766 ± 615
	--	6.01 ± 2.52	2.44 ± 2.02	2.53 ± 0.75	3.09 ± 1.99

### Relative Contributions of Macronutrients

**Fig 2:**
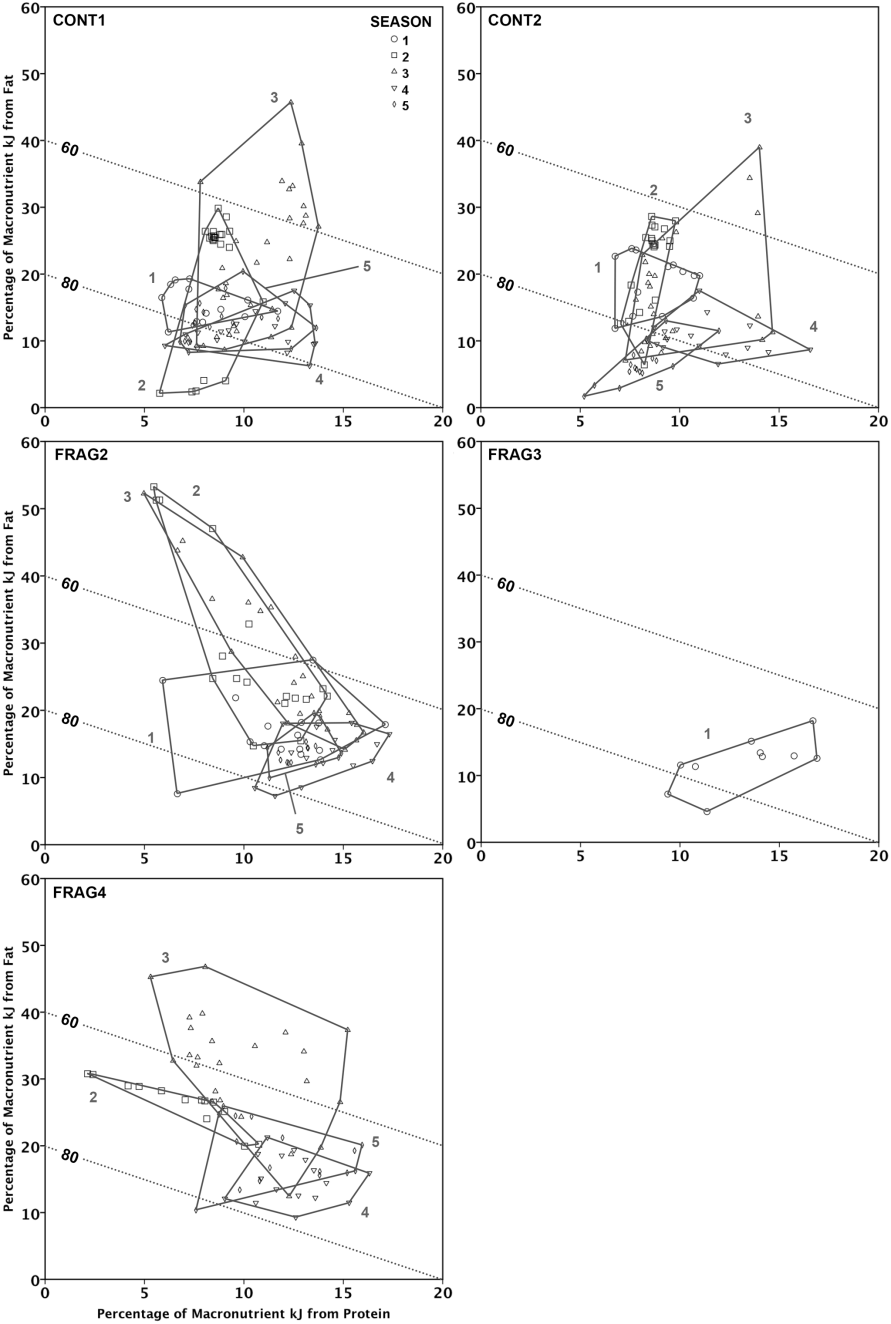
Macronutrient balancing in sifaka groups across seasons at Tsinjoarivo, Madagascar. Right-angle mixture triangle (RMT) plots showing the proportional contribution of protein, fat and carbohydrates to macronutrient-derived energy intakes for five sifaka groups at Tsinjoarivo, Madagascar across five seasons (1, 4 and 5 represent the lean season). Fat and protein are represented on the y- and x-axes, respectively, and carbohydrate is represented by an implicit axis Z. For clarity, each plot has two “isolines” representing 60% and 80% carbohydrates, shown with dotted lines.

When considering only easily-digested energy (protein, fat, and carbohydrates; excluding fiber), FRAG groups tended to have lower contributions from carbohydrates and higher contributions from protein (and, except for FRAG4, from fat; Fig 2, Table 4). For CONT groups, lean season diets (seasons 1, 4 and 5) have 5–25% of kJ from fat, 5–15% of kJ from protein, and 70–90% of kJ from carbohydrates. In the abundant season (especially season 3), groups derive more energy from fat (10–50%), with a corresponding decrease in energy derived from carbohydrates (as low as 50%), but little change in the contribution of protein. In other words, the abundant season sees an expansion mainly along the y-axis.

**Table 4:** Relative contribution of macronutrients to daily dietary intakes for five sifaka groups at Tsinjoarivo, Madagascar.

Group	Percentage contribution to macronutrient kJ (Mean ± SD; available protein, fat and TNC; excluding fiber) of:			AP:NPE (NPE includes fat and carbohydrates, including fiber) of Daily Diet	
	Protein	Fat	Carbohydrate	Mean ± SD	CV
CONT1	9.5±2.1	17.0±8.4	73.6±9.2	0.096±0.023	24.1%
CONT2	9.4±2.1	15.4±8.0	75.2±8.5	0.093±0.024	26.1%
FRAG2	12.1±2.8	21.0±10.8	66.9±9.2	0.125±0.031	24.9%
FRAG3^1^	13.3±2.7	12.0±3.8	74.8±6.0	0.133±0.028	21.0%
FRAG4^1^	10.3±3.3	23.9±8.9	65.8±7.7	0.104±0.035	33.5%
TOTAL	10.3±2.8	18.7±9.5	70.9±9.5	0.105±0.031	29.6%

^1^ Groups FRAG3 and FRAG4 are not represented across the entire annual cycle

FRAG groups occupy similar dietary space to CONT groups during the lean and abundant season, but with two differences. First, there is more variation along the x-axis: during the lean season, protein is higher than in CONT groups (10–18%), but this contribution declines in the abundant season (2–15%). Second, the abundant season diet has a similar increased contribution from fat, but FRAG2’s highest fat days saw a marked decrease in protein contribution (leaning to the left).

### Contribution of Protein to the Diet

**Fig 3:**
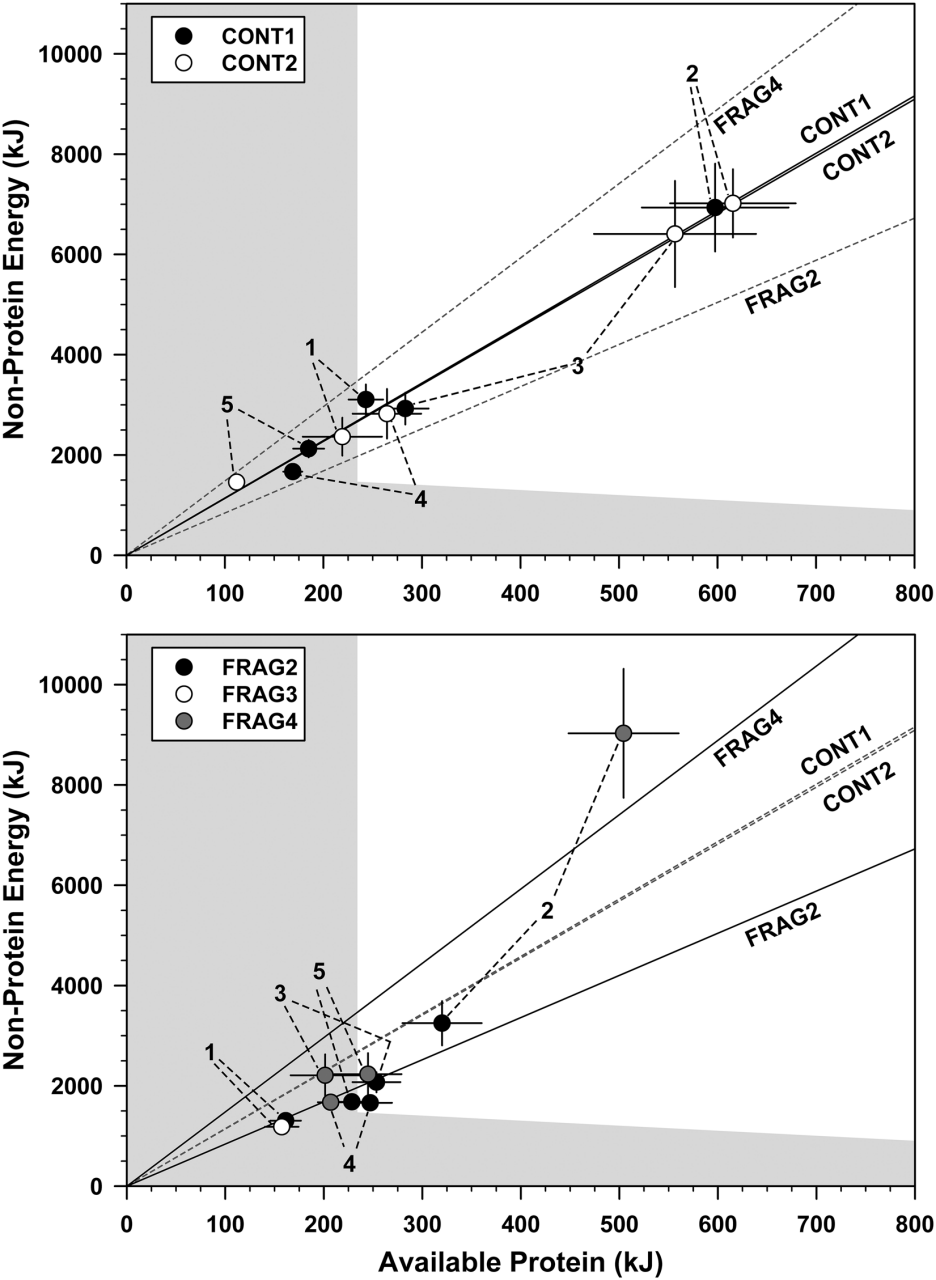
Seasonality in the contribution of protein to the diet in sifaka groups at Tsinjoarivo, Madagascar. Bivariate plots showing absolute seasonal averages (daily intakes ± SE) of protein (x-axis) and non-protein energy (fat + carbohydrate + NDF; y-axis), both expressed in kJ, for five sifaka groups at Tsinjoarivo, Madagascar. The fit line represents a linear regression forced through the origin as a visual approximation of each group’s nutritional rail (with grey dashed lines indicating rails for groups in the other panel). The shaded area represents nutritional space (i) left of a vertical line representing estimated minimum protein requirement of 2.8 g●BM_kg_^-1^●day^-1^, equivalent to 234 kJ for a 5-kg sifaka, and/or (ii) below an oblique line representing 500 kJ●BM_kg_^-0.762^●day^-1^, equivalent to 1703 kJ for a 5-kg sifaka (see discussion for details on requirements).

Sifakas had a highly consistent AP:NPE ratio: across all groups, the ratio was 0.105 (corresponding to 9.47% of daily energy intake derived from protein; Table 4; manifested as aligning along a single “nutritional rail” in Fig 3). LMMs revealed significant effects of group and time period, and the interaction (Table 2). The effect of time period is largely driven by FRAG groups’ abundant season reductions (FRAG2 seasons 1–5: 0.122, 0.104, 0.122, 0.147and 0.135 respectively, FRAG4 seasons 2–5: 0.068, 0.097, 0.127, 0.120); CONT groups’ variation was much less.

The 76 sifaka foods for which macronutrient and energy densities are known showed a higher mean and variation compared to daily diets (mean: 15.1; SD: 12.5; range 0–79.7; CV: 83.3%). The most important foods also showed considerable variation in AP:NPE ratio (Table 5).

**Table 5:** Macronutrient concentrations of 8 most important foods utilized in CONT and FRAG sites, including the proportion of available energy derived from protein; full nutritional profiles in Irwin et al. [20].

Food	% Feeding time	% Available Protein	% TNC	% Fat	% NDF	AP:NPE
**CONT groups:**						
SD, *Salacia madagascariensis*	7.2	4.2	45.1	2.0	43.5	0.070
YL, *Maesa lanceolata*	6.9	11.5	52.1	5.7	29.5	0.184
YL, *Bakerella clavata* var. 1	6.4	1.0	37.3	0	54.2	0.023
YL, *Garcinia* sp. “Voamalambotaholahy”	5.9	8.7	50.2	3.3	34.8	0.142
SD, *Cryptocarya* sp. “Tavolo”	5.5	na	na	na	na	na
FSD, *Syzygium* sp. 2 “Rotra mena SL”	5.4	2.6	57.2	2.0	35.8	0.039
YL, *Bakerella clavata* var. 2	3.4	3.8	50.0	na	41.3	0.061*
BD, *Bakerella clavata* var. 2	3.4	4.7	51.1	6.3	33.9	0.066
**FRAG groups:**						
YL, *Maesa lanceolata*	9.7	11.5	52.1	5.7	29.5	0.184
BD, *Bakerella clavata* var. 2	9.6	4.7	51.1	6.3	33.9	0.066
YL, *Bakerella clavata* var. 1	8.7	1.0	37.3	0	54.2	0.023
URFSD, *Bakerella clavata* var. 1	5.6	0	20.1	25.3	46.6	0
YL, *Symphonia* sp. 1 “Kimba tenany”	3.9	10.3	33.9	3.4	50.0	0.217
FSD, *Schefflera vantsilana*	3.9	7.5	49.5	6.8	32.5	0.103
YL, *Embelia concinna*	3.4	8.1	45.2	na	42.5	0.144*
F, cf. *Drypetes madagascariensis*	2.9	13.2	55.0	0.2	28.0	0.215

*Not all YL samples were analyzed for fat; when not analyzed, the average value for YL samples was substituted (1.8%)

### Amounts-based Analysis of Daily Macronutrient Intakes

Although proportional contributions can provide important insights, it is important to remember they are superimposed on diet-wide changes in sifakas’ dry mass intake. The relative invariance of the AP:NPE ratio (especially in CONT groups) combined with lean season decreases in consumption cause all groups’ seasonal AP and NPE averages to align along a single “nutritional rail” in bivariate space, meaning that AP:NPE was relatively invariant (Fig 3). The fact that the data were spread along the nutritional rail suggests that amounts of macronutrients eaten varied more than did the ratios. All groups occupy similar nutritional space in the lean season, but CONT groups progress much farther from the origin in the abundant season than FRAG groups.

## Discussion

### Ecological Context and Intergroup Differences

It is important to remember the groups represent a gradient of disturbance rather than a strict continuous forest vs. fragment dichotomy [41]. CONT groups occupy the least disturbed habitat, as evidenced by basal area values of 40–45 m^2^/ha. FRAG3 had the lowest basal area (9 m^2^/ha) and it is notable that this group spent more time feeding than others, yet ranked lowest in mass ingested, frugivory, and all intakes except NDF. These poor nutritional outcomes may have contributed to the otherwise unexplained death of both group members.

The two remaining groups were intermediate in disturbance; FRAG2 had the second-most disturbed habitat (17 m^2^/ha), while FRAG4’s habitat had 23 m^2^/ha. They were similar in many aspects of foraging and intakes, but FRAG4 was able to achieve greater frugivory and higher intakes in some seasons. Season 2 is an outlier; FRAG4’s high intakes derive from an especially good crop of “Tavolo” seeds (*Cryptocarya* sp.), which was the top food in CONT groups that season (CONT1: 18.9% of feeding time, CONT2: 24.7%), the number 3 food in FRAG4 (16.6%), but only the tenth-most consumed food in FRAG2 (3.2%). Tavolo is a valued timber tree that has been exploited locally, and its abundance declines with habitat disturbance; it remains abundant in CONT2 (3944 m^3^/ha crown volume) and CONT1 (1811 m^3^/ha), and is less abundant in FRAG4 (1648 m^3^/ha), FRAG2 (970 m^3^/ha) and FRAG3 (445 m^3^/ha; [41]).

More generally, it is striking that many aspects of foraging and intakes align with the ordering of groups based on basal area. A decreasing basal area per hectare seems to correlate with increased feeding time, yet decreased frugivory, mass intake, and energy and nutrient intakes, with especially divergent intakes in the abundant season. Although more replication of groups would be desirable, these data suggest that it might be the habitat disturbance itself driving these changes. This is consistent with the evidence that sifakas, historically thought of as folivores, consume fruit in relation to its abundance [29]. Thus, sifakas in disturbed habitat experience little change from the abundant season to the lean season (when all groups eat mainly flowers and leaves), but find it harder to be the frugivore they apparently prefer to be in the abundant season—and the CONT groups’ nutritional “lift” in the abundant season illustrates why this is desirable.

### Adequacy of the Diet and Comparisons with other Primates

Primate energy requirements are poorly known, though lemurs’ mass-specific requirements may be lower than those of other primates [46]. King [47] reported three captive lemurs (*Eulemur fulvus*, *E*. *mongoz*, and *Lemur catta*) had energy intakes of 361–514 kJ●BM_kg_^-0.75^●day^-1^ (though they used a slightly different scaling factor; values scaled to 0.762 should be roughly 1% lower and therefore roughly comparable). Sifakas’ daily intake averaged 1049 kJ●BM_kg_^-0.762^●day^-1^, with seasonal averages ranging from 407 to 2799. Daily values went as low as 202 kJ●BM_kg_^-0.762^●day^-1^ and 10 days had values less than 300. Sifakas likely experience energy surplus in the abundant season, but intakes at or below maintenance requirements in the lean season. Deficits are likely more pronounced once the added requirements of adult females (reproduction) and immatures (growth) are considered, but these costs are not well-quantified [43].

In terms of protein, adult non-human primates are thought to require 1.8–2.8 g●BM_kg_^-1^●day^-1^, [43], equivalent to 9–14 g/day (151–234 kJ) for a typical adult *P*. *diadema* (5 kg). Sifakas seemed to consume protein in surplus in season 2, though FRAG2’s intakes were much lower (3.98 g●BM_kg_^-1^●day^-1^) than other groups (6.01–7.61 g●BM_kg_^-1^●day^-1^). Lean season intakes were low, averaging 2.5 g●BM_kg_^-1^●day^-1^, with a lowest recorded value of 0.59 and 55 of 188 days falling below 1.8. Though few data are available for lemurs, juvenile primates are thought to require 2.5–7.3 g●BM_kg_^-1^●day^-1^ [43]; as the fastest-growing juveniles in this study were 2- and 3-year olds, ranging from 4000 to 4462.5 g, this translates to roughly 10–33 g/day (167–489 kJ/day). Together, these results suggest Tsinjoarivo sifakas (especially juveniles) experience lean season protein stress.

Few comparative primate studies are available, and comparisons should be viewed cautiously due to methodological differences; however, sifaka energy intakes seem similar to or higher than estimates for other primates. Aye-ayes (*Daubentonia madagascariensis*) had intakes of 548–721 kJ●BM_kg_^-0.762^●day^-1^ (assuming body mass of 2.46 kg) [48], while spider monkeys consumed 403 kJ●BM_kg_^-0.762^●day^-1^ [25]. Mountain gorillas had higher intakes: 680 kJ●BM_kg_^-0.762^●day^-1^ for adult males, 1024 for adult females and 1111 for juveniles [7]. However, it is important to remember these are annual averages; as the fluctuation seen in sifakas is extreme, seasonal energy stress may be more pronounced.

In terms of available protein, sifaka intakes were similar to gorillas (2.98 g●BM_kg_^-1^●day^-1^ for adult males, 5.25 for adult females) [7, 49], but varied more across seasons. In contrast, spider monkey intakes were quite low (1.4 g●BMkg^-1^●day^-1^) [25] and consistent across seasons. This suggests different macronutrient regulatory strategies, in which gorillas eat protein in surplus [28] and spider monkeys strictly regulate daily protein intakes at a value close to its limiting value across seasons. Sifakas are the most variable: their lean season values dip as low as spider monkeys but their abundant season values exceed those of gorillas. This may introduce severe seasonal constraints, especially on juveniles’ growth and females’ reproductive investment.

### Nutritional Geometry of Habitat Change

Assuming that CONT groups represent typical sifaka nutritional inputs, it appears that the groups most impacted by habitat change (FRAG2, FRAG3), show altered foraging patterns year-round, with more time spent feeding, yet the amount of food ingested does not reflect this increased effort. During the lean season, all groups converged on similarly low daily intakes, which seem to be near or below the lowest acceptable intakes of energy and protein. However, the CONT groups experienced a marked abundant season “lift” in feeding time, frugivory, mass ingested, and energy/macronutrient intakes, most particularly fat. CONT groups’ energy and protein intakes show a three- to four-fold difference between lean and abundant seasons. FRAG groups (especially FRAG2 and FRAG3) also increased feeding time and frugivory in the abundant season but failed to realize the “lift”. In other word, their fallback foods seem to have survived habitat disturbance unscathed, while abundant season resources have been decimated. This is consistent with previous reports that CONT and FRAG groups converge on the same foods in the lean season, concentrating on leaves and flowers of the mistletoe *Bakerella clavata*, and diverge in the abundant season, when FRAG groups concentrate on *B*. *clavata* fruits while CONT groups concentrate on fruits of large trees now rare in fragments due to overexploitation [29].

The average proportion of sifakas’ daily macronutrient intake (expressed in kJ) derived from protein (9.47%) was lower than in mountain gorillas (19–30%) [28], but almost identical to spider monkeys (9.45%) [25]. Mountain gorilla diets are at the middle and upper parts of the recommendations for human diet (10–35% of energy) [50], while sifakas and spider monkeys fall just below this range. Perhaps more importantly, sifaka variation neither resembles spider monkeys (maintaining near-constant protein intake while NPE intake varies) [25], nor gorillas (maintaining near-constant NPE intake while protein intake varies) [28]. Instead, an as-yet undocumented third pattern is seen: protein and NPE intakes are tightly correlated (Fig 3), meaning that the percentage of dietary energy that could potentially be derived from protein (and the AP:NPE ratio) is highly conserved across seasons despite a large difference in mass and energy intakes. This mirrors the balancing strategy seen in a 30-day study of a single chacma baboon [51]. Thus, seasonal differences in sifaka protein and NPE intakes follow a single nutritional rail, whereas spider monkeys track a vertical line and gorillas a horizontal line.

### What is the Sifaka “Strategy”?

An interesting question is whether a macronutrient regulation “strategy” can be inferred from this pattern, in the same way that frugivorous spider monkeys seem to “maintain minimum protein intake” and primarily folivorous gorillas seem to “maintain minimum NPE intake”. The wide range of AP:NPE ratios in available foods across both seasons (Table 5) means that sifakas, in theory, should be able to pursue a gorilla or spider monkey strategy; most of the bivariate space depicted in Fig 3 is available to them. It is possible that daily and seasonal intakes converge on the observed AP:NPE ratio by chance, perhaps in part because other properties of food such as PSMs force sifakas to spread foraging effort across many species.

If sifakas deliberately choose foods so as to maintain their AP:NPE ratio, this “strategy” would not fit a simple optimization or maximization rule, but rather a balancing mechanism. If the lean season is thought to induce a rule of compromise when nutritional targets cannot be attained, one must start by considering the nature of the lean season diet. Sifakas respond to reduced fruit availability in the lean season by shifting to young leaves and flowers and increasing dietary diversity [29], and during this lean season their overall mass ingested per day drops to roughly 40% of abundant season intakes [20]. It remains unknown why sifakas do not simply eat more food (indeed they reduce their feeding effort). Although Tsinjoarivo is an evergreen humid forest, the lean season sees the lowest availability of all food types [29] and key foods may be either extremely low value (e.g., tiny mistletoe flowers) or chemically defended (e.g., leaves). Irwin et al. [20] suggested that PSM content in leaves was the reason sifakas did not increase lean season foraging effort, following the detoxification limitation hypothesis [9]; in this case PSMs may force sifakas to increase dietary diversity to avoid overload of particular toxins, thereby forcing them into an intermediate AP:NPE; in other words balancing foods to reduce overall toxicity takes precedence over actively managing macronutrient ratios.

**Fig 4:**
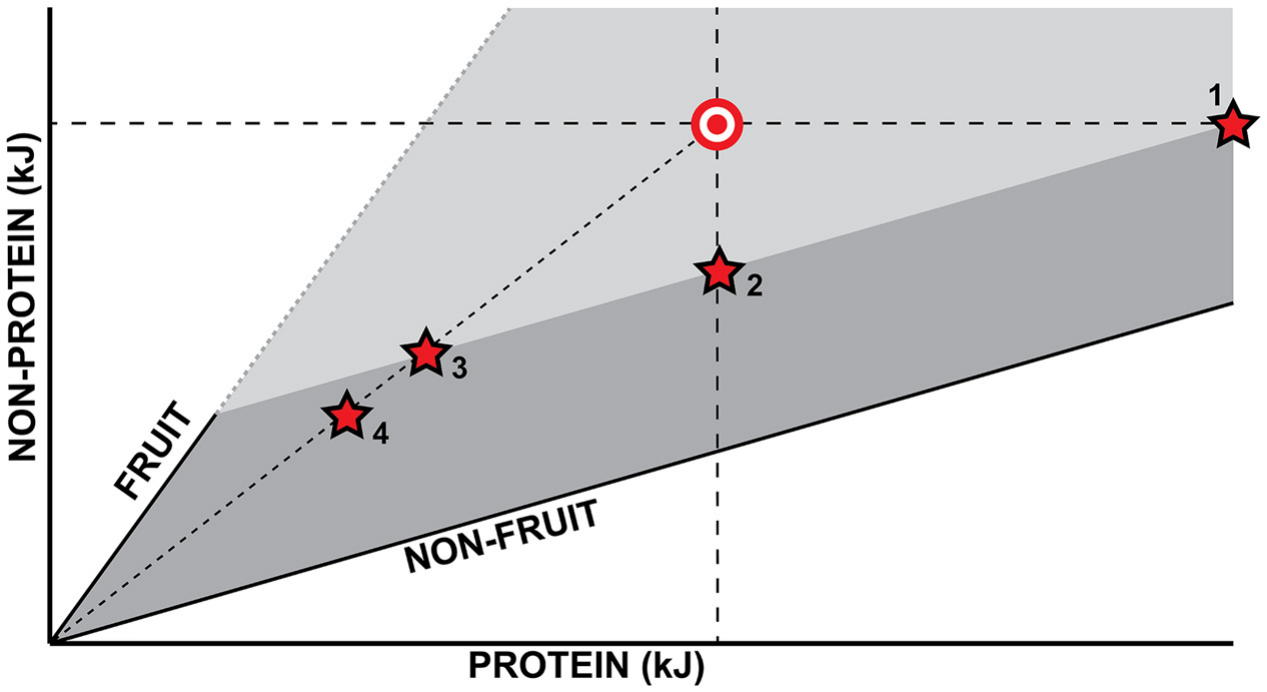
Schematic depicting different lean season “rules of compromise” when nutritional target (bullseye) cannot be met. The abundant season target is assumed to be a balanced target achieved using fruits (lower AP:NPE ratio, steeper nutritional rail) and non-fruit foods (higher AP:NPE ratio, shallower nutritional rail), and one of the major constraints of the lean season is modeled as reduced fruit availability (solid component of fruit nutritional rail). Lower fruit availability in the lean season contracts the available nutritional space from all grey areas to the darker grey area. Mountain gorillas (1) overeat protein-rich non-fruit foods to meet NPE target; spider monkeys (2) fall short on non-protein energy target but meet protein target. Sifakas might be constrained by both fruit availability and a need to preserve the abundant season’s AP:NPE ratio (3), or intakes might be further limited by plant secondary metabolites (PSMs), which limit non-fruit intakes and would explain low lean season mass intakes (4). Note that different species are likely to differ in their “starting points” (a common starting point is used for simplicity of representation); for example, mountain gorillas’ abundant season “targets” are relatively higher in protein than either spider monkeys or sifakas.

In short, sifakas may not have a simple rule of compromise in the way that spider monkeys and gorillas do (positions “1” and “2” in Fig 4). One possibility is that sifakas actively forego a chance to meet at least one of the two targets (protein) through non-fruit foods, in order to actively preserve constant macronutrient ratios (position “3”). We find it more likely that the combination of low fruit availability and the properties of lean season non-fruit foods (e.g., PSMs) interact to severely restrict the attainable mass intakes for all food types (position “4”), which causes universally low intakes and a situation in which no compromise can be found. It is also important to note that one of the main sources of lean season leaves was mistletoe (*Bakerella clavata*), which had extended phenology and greater lean season availability than most trees, but unusually low protein content (Table 3); this likely exacerbated protein shortfalls.

### Implications for Lemur Ecology and Conservation

Sifakas mitigate serious lean season nutritional deficits by overeating both protein and NPE during the abundant season, perhaps to build fat and muscle tissues to draw on during the lean season. It has been suggested that Madagascar presents an unusual resource environment for herbivores, either in terms of diet quality [52], or more pronounced seasonality and lower resource predictability [53]. Madagascar’s herbivores seem to have responded via extreme birth seasonality [54] and seasonal mass changes [55], and are well-adapted to surviving periods of food scarcity [3, 56]. However, to our knowledge this study is the first to present quantitative nutritional data suggesting that lemurs experience seasonality in ways fundamentally different from other primates. The low lean season protein intakes reported here suggest that immature sifakas may fail to sustain growth in lean seasons. Although logistically challenging, it would be interesting to quantify mass change across seasons in adults [57] or determine whether juveniles’ growth is slowed or arrested in lean seasons [58]. In terms of reproduction, the observation that lemur species concentrate weaning in February/March, in the abundant season [59], is consistent with the idea that seasonality has placed high selective pressures on lemur life histories and may also explain why lemurs and other strepsirrhines have lower fetal growth rates than haplorrhines [60]. This is especially true for larger-bodied lemurs like sifakas, for whom late gestation, birth and the first few months of lactation occur in the lean season (April-October). Future research should address how mothers cope with nutritional deficits, especially investigating nutrient storage (capital breeding), energy conservation, slower-than-expected fetal or infant growth rate, and catch-up growth, including accelerated dental development [56]; these constraints may also be linked to the evolution of female dominance [59].

In terms of conservation, we suggest that the lean season may be the critical time for reproductive disruption and/or mortality, but the more important consequence of habitat change is manifested in the abundant season. We speculate that lemurs may be well-adapted to lean seasons if they can replenish body stores during abundant seasons. Conservation efforts should therefore focus on maintaining key abundant season foods.

This study supports the idea of qualitative differences between lemurs and haplorhine primates. For example, sifakas’ absolute intakes of all macronutrients and energy reach (very) low points in the lean season, while gorillas experience less seasonal variation in intakes, and a distinct trade-off, with highest carbohydrate intakes in the fruiting season and highest protein and fiber intakes in the non-fruiting season [7]. However, other comparisons contradict this, such as the extreme seasonal variability in orangutan intakes [8, 23, 61]. More sampling, especially studies that quantify intake, feeding effort and phenology across seasons, is necessary to investigate other sources of variation (environment, phylogenetic inertia, body size, PSMs).

An improved understanding of macronutrient balancing in wild herbivores would yield several benefits. First, it will help in understanding how food availability naturally limits populations’ growth and persistence. On a regional level, the positive correlation between protein:fiber ratios in foliage and the local density of folivores suggests that protein is a limiting factor in population growth and maintenance [62–65]. Second, it will help in preventing extinctions in disturbed landscapes, by identifying habitat management strategies that minimize nutritional disruptions. Finally, it has the potential to contribute to understanding the evolution of social systems [66], particularly differentiating the ideas that competition during food shortage may have a disproportionately high proximate impact on survival and reproduction, but that abundant seasons may be ultimately important in replenishing body condition. Thus, emergent social strategies may be a complex product of a subset of foods and/or seasons rather than reflecting a species’ broader feeding ecology.

## Supporting Information

S1 Table Daily foraging and nutrient intake variables for five sifaka groups at Tsinjoarivo, Madagascar (2006–2007). (XLSX)
